# Adaptive Detection of Direct-Sequence Spread-Spectrum Signals Based on Knowledge-Enhanced Compressive Measurements and Artificial Neural Networks

**DOI:** 10.3390/s21072538

**Published:** 2021-04-05

**Authors:** Shuang Zhang, Feng Liu, Yuang Huang, Xuedong Meng

**Affiliations:** 1 College of Electronic Information and Optical Engineering, Nankai University, Tianjin 300350, China; 2120190332@mail.nankai.edu.cn (S.Z.); 1711088@mail.nankai.edu.cn (Y.H.); 1711116@mail.nankai.edu.cn (X.M.); 2 Tianjin Key Laboratory of Optoelectronic Sensor and Sensing Network Technology, Nankai University, Tianjin 300350, China

**Keywords:** DSSS, compressive detection, mutual information, adaptive detection, artificial neural network

## Abstract

The direct-sequence spread-spectrum (DSSS) technique has been widely used in wireless secure communications. In this technique, the baseband signal is spread over a wider bandwidth using pseudo-random sequences to avoid interference or interception. In this paper, the authors propose methods to adaptively detect the DSSS signals based on knowledge-enhanced compressive measurements and artificial neural networks. Compared with the conventional non-compressive detection system, the compressive detection framework can achieve a reasonable balance between detection performance and sampling hardware cost. In contrast to the existing compressive sampling techniques, the proposed methods are shown to enable adaptive measurement kernel design with high efficiency. Through the theoretical analysis and the simulation results, the proposed adaptive compressive detection methods are also demonstrated to provide significantly enhanced detection performance efficiently, compared to their counterpart with the conventional random measurement kernels.

## 1. Introduction

In this era, the direct-sequence spread-spectrum (DSSS) has become one of the most widely used spread-spectrum (SS) techniques in the wireless secure communications [1]. In DSSS, the baseband digital code streams are spread into a much wider band through the modulation with pseudo-noise (PN) sequences. Due to the wide bandwidths, the DSSS signals usually keep a low power-spectrum density and are hidden under the channel noise. For cooperative receivers, the PN sequences are exactly known. Thus, the baseband signals can be directly recovered through demodulation. In contrast, for a non-cooperative receiver without the exact knowledge of the PN sequence, the DSSS signals appears merely noise [2]. Moreover, without precise knowledge of the PN sequence, the conventional non-cooperative receivers must operate at a high sampling rate to catch the signal, according to Nyquist sampling theorem. This significantly increases the system cost and sometimes makes the system impossible to be implemented.

The detection of the DSSS signals is the prerequisite to the following signal processing and information extraction steps [3,4]. It has been intensively studied ever since the beginning years of the DSSS technique. To detect the DSSS signals, many methods have been proposed, such as energy-based, analysis of fluctuation based on second-order statistics, dirty template-based, etc. The most commonly used method among them is the energy-based detection [5], which is easier and relatively less expensive to be implemented [6,7]. However, due to the wide bandwidths of the DSSS signals, high sampling rates are required in those methods to capture the entire spread-spectrum, which usually brings a burden on the hardware cost.

In last decade, the compressive sensing (CS) theorem was rendered [8,9], which provided perspectives on sufficient sampling on image and communication signal processing techniques [10,11,12]. Motivated by the CS theorem, many compressive signal detection methods were proposed, such as sparse signal reconstruction-based methods. However, only random measurement kernels were proposed in most of these methods. In [13], the measurement kernels were proposed to be designed based on recursive information optimization. However, the high time and computational costs determined that the method was not feasible in the adaptive measurement and detection scenarios.

Presently, with the rapid development of the computer technologies, especially the parallel computing, the artificial neural network (or neural network) and the deep learning [14] techniques have been widely used in the area of signal processing, such as topics on biomedical and civil engineering [15,16]. The well-trained neural networks can efficiently extract the features of the signals, and are proved to have good performance in pattern recognition, signal parameter estimation, prediction, etc. Therefore, it is possible to improve the detection performance and adaptability through the neural networks.

In this paper, we propose methods to detect the DSSS non-cooperatively and adaptively based on knowledge-enhanced compressive measurements and artificial neural networks. The measurements are done with compressive rates to reduce the costs of sampling process and the detection decisions are made based on measurement energy thresholding. The detection task-specific information (TSI) quantitative analysis with the signal posterior probability updates is introduced in the adaptive measurement design to improve the detection accuracy. To greatly improve the efficiency of the algorithm, the artificial neural networks are trained based on the TSI optimization. The resulting neural networks can take the posterior probabilities of the signals from the Bayes updates as the inputs and directly give the adaptively designed measurement kernels.

Our work makes several novel contributions:

(1) Compared to the existing compressive detection methods, the proposed methods enable an adaptive compressive measurement framework, where the measurement kernels can be flexibly adjusted to track the DSSS signals without the exact prior knowledge of the PN sequences in the detection.
(2) To ensure the gain of the detection accuracy, the quantitative information analysis from the previous measurements is implemented in the following adaptive measurement matrix design, ensuring the gradually increased correlation to the most probable signals.
(3) Through the effective combination of knowledge-enhanced compressive measurement with TSI optimization and the artificial neural network techniques, the compressive measurement matrix can be designed in an both adaptive and efficient manner. Compared to the recursive measurement kernels directly optimized based on quantitative information analysis in the literature, the artificial neural networks are trained based on TSI optimization off-line and implemented repeatedly and efficiently in the online adaptive measurement kernel design, which not only improves the adaptability, but also saves a lot of detection time.

From the aspect of the signal processing systems, with the proposed method, both the efficiency of the adaptive measurement system and the adaptability of the neural network-based system are achieved.

The remainder of the paper is organized as follows: In Section 2, the existing methods in DSSS signals detection are briefly discussed as the background of this paper. Then in Section 3, the framework and the principle of the proposed DSSS signals adaptive compressive measurement and detection methods are introduced. In Section 4, the design and the implementation of the artificial neural networks in the proposed adaptive measurement and detection framework are detailed. Then in Section 5, the proposed methods are evaluated and discussed through the theoretical analysis and the simulations with DSSS signals. Finally, the conclusions are drawn in Section 6.

## 2. Related Works

With the rapid development of the communication technology, spread-spectrum communication has become an important way in the modern communication system. DSSS communication system has been widely used in the military and civil communication domain. From the electronic countermeasure perspective, to intercept or interfere signals that may be transmitted in the DSSS mode, it is necessary to first detect whether there is a DSSS signal present in the wireless channel, before finally recovering the information contained in the signal. Therefore, the detection of the DSSS signals is indispensable in the entire DSSS signal reception process.

Due to the importance of the DSSS signal detection step, a lot of research has been done in this area with a series of detection methods proposed, which can be basically classified as non-compressive and compressive detection methods. In the following part of this section, the two types of methods are introduced as the background of the DSSS signal detection techniques.

### 2.1. Non-Compressive Detection Methods

As is implemented in conventional methods of the signal processing, the signals were sampled according to the Nyquist sampling rate to capture their entire spectrum and avoid aliasing. Before the CS theory was rendered, researchers proposed many non-compressive detection methods, such as energy-based detection methods [17], auto-correlation-based detection methods [18,19] and spectrum-based detection methods [20,21,22], etc. These methods are introduced in the remainder of this subsection.

Back to the early 1960s, H. Urkowitz proposed the energy-based detection method [17], where the detection was done based on the fact that the energy of noise is small than the total energy of the signal and noise. By calculating the energy of the received signal and selecting an appropriate threshold, the DSSS signals can be detected in the DSSS signal present case. In the existing non-compressive detection methods, the energy-based detection method is the simplest and least expensive, and thus is commonly used.

The autocorrelation-based detection methods [18,19] were first used to detect the frequency hopping spread-spectrum (FHSS) signals, and later researchers extended it to the detection of the DSSS signals. These detection methods perform auto-correlation operation on the received signal based on the difference between signal and noise in the auto-correlation domain. Then the correlative peaks are implemented to detect the DSSS signals. Burel et al. rendered a detection method of fluctuation analysis based on second-order statistics, which was done by dividing the received signal into analysis windows, calculating second-order statistics on each window, and then using the results to compute the fluctuations [23]. However, as a drawback of all these methods, the correlative peaks or the second-order statistics are still not easy to be extracted if the signal-to-noise ratio (SNR) is low, which makes these detection scheme not viable in low SNR scenarios.

The spectrum-based detection methods (time-frequency analysis-based [20,21], short-time Fourier transform-based [22], etc.) model the DSSS signals as periodic stationary, and perform the detection decisions in the transform domain. These methods have good detection performance for the non-periodic stationary signals or the low SNR environment, but suffer from cross-interferences. Moreover, the computational complexity was high, which results in slow detection speed and difficulty in real-time implementation. In 2019, Lee and Oh proposed to implement a dirty-template-based scheme in the detection of SS signals [24], which could also be implemented in the DSSS signals detection. This detection method was done by calculating the cross-correlation between the template and the received signals in the frequency domain. However, the ‘dirty’ template was obtained from the received signal in frequency domain, which would make the template difficult to be obtained in the low SNR scenarios.

Although various methods based on non-compressive sampling were rendered in the past few decades, a common shortcoming exists within them: The high sampling rates are required to capture the entire spectrum of the DSSS signals, resulting in expensive sampling and signal processing hardware. Especially in this cases of ultra-band DSSS signals, these methods may become infeasible. Moreover, there is a lack of adaptability in these methods, leading to a constraint on the further improvement of their detection performance.

### 2.2. Compressive Detection Methods

In 2006, the compressive sensing (CS) theorem [8,9] was rendered by Candes et al. and Donoho. In contrast to the conventional Shannon-Nyquist sampling theorem, the CS theorem states that a signal can be recovered from much lower number of its linear projections (i.e., low measurement rates), if the signal can be sparsely represented on a transform or a dictionary. The signal recovery can be done by solving non-linear optimization problems respective to its sparse representation. Motivated by the measurement rates in this theorem, a series of DSSS signal detection methods based on CS have been proposed.

Most of the existing CS-based DSSS signal detection methods were proposed based on random measurement kernels and CS recovery methods. Some of these methods retained the information carried by a signal, which could be sparsely represented based on a transform or a dictionary [25,26,27,28,29]. Others cooperatively detected the signal based on the signal reconstruction or the expression of the original signal [30,31]. However, the reconstruction algorithms usually require high computational complexity, which greatly affect the computational efficiency of the algorithms, especially in the online signal detection scenarios.

Although most of the literature on CS used random measurement kernels, Gu et al. [32] and Neifeld et al. [33] illustrated that the signal recovery accuracy could be improved, if the compressive measurement kernels were designed using prior knowledge of the signal. More recently, Liu et al. proposed non-cooperative compressive DSSS signals detection methods [13]. In contrast to most of the existing literature in area of the CS-based DSSS signal processing that included an intermediate step of signal or information recovery, the detection decision was directly made from the compressive measurements. Besides random measurement kernels, the designed measurement kernels were also proposed based on the prior knowledge of the signals and the quantitative information optimization [33]. However, as the measurement kernel optimizations were conducted using a recursive method and could take an extremely long time, the measurement kernels in Liu et al. [13] had to be designed prior to the measurement procedure and could not be used in the adaptive regimes.

In this paper, we propose methods to detect adaptive DSSS signals based on knowledge-enhanced compressive measurements and artificial neural networks. With the compressive measurements, the hardware burden caused by the non-compressive detection methods are solved. The detection decisions of the DSSS signals are made by the observation of the measurement energy, which is easier and less expensive than most of the compressive measurement-based detection methods. Moreover, with posterior knowledge of the signal updated and the implementations of the artificial neural networks, the measurement kernels are designed adaptively and efficiently with the quantitative TSI optimized, leading to improved detection performance.

## 3. The Framework and Principle of the Adaptive Compressive Measurement and Detection of the DSSS Signals

**Figure 1:**
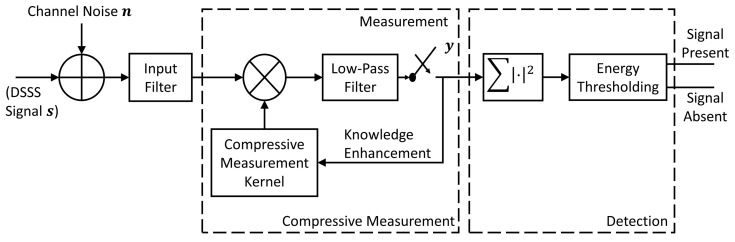
The Adaptive Compressive Measurement and Detection Framework.

The proposed compressive measurement and detection framework is shown in Figure 1. In the measurement step, the received signal is first preprocessed by a band-pass filter to remove frequency components outside the spectrum of interest. The preprocessed signal is then multiplied by the compressive measurement kernels and passed through a low-pass filter, which works as an integrator. The filtered result is sampled with a sampling rate that is much lower than the Nyquist sampling rate indicated by the DSSS spectrum. The sampling results form the measurement vector. The measurement vector is analyzed based on Bayes rule and the analyzed results are used in the adaptive measurement kernel design for the following measurements, as the knowledge enhancement in the compressive measurement procedure. Finally, in the detection step, the energy of the measurement vector is calculated and thresholded to determine if the DSSS signal is present.

In this paper, we focus on the non-fading communication channels and the signal detection using the framework in Figure 1 can be formulated as a decision from two hypotheses:

(1) $$\begin{array}{ccc} H_{0}:\;y=An & \; & H_{1}:\;y=A\left(s+n\right), \end{array}$$

where $H_{0}$ and $H_{1}$ represent the signal absent and signal present hypotheses of the DSSS signal, respectively. A is the compressive measurement matrix, s is the DSSS signal at the receiver in the signal present case, n is the channel noise and y is the compressive measurement vector. The compression ratio (CR) of the system is defined by the ratio between the number of Nyquist samples with respect to the spread bandwidth and the number of compressive measurements in a given time period. For the system in Figure 1, the measurement matrix is block-diagonal, where each single block is a row vector. The coefficients in each row block of the measurement matrix form the measurement kernel of the corresponding measurement.

In this paper, we take the phase-shift-keying (PSK) DSSS signals as examples, which can be represented as:

(2) $$s\left(t\right)=c\left(t\right)\times d\left(t\right)e^{j(2\pi f_{c}t+\phi )},$$

In Equation (2), c(t) is the baseband PSK signal, d(t) is the binary-valued (1 or −1) periodic signal modulated by the PN sequence, $f_{c}$ is the carrier frequency and ϕ is the initialized random phase.

In this paper, we model the wireless channel as additive white Gaussian noise (AWGN) channel over the DSSS spectrum with the noise variance of $\sigma_{n}^{2}$. Let us consider that the rows of measurement matrix are normalized to unit energy. According to the noise folding theory [34], the measurement vector would become a zero-mean circularly symmetric complex random vector with the variance of $\sigma_{n}^{2}$. Then in the signal absent case, the theoretical probability density function (PDF) of the energy in a measurement vector y at the length *M* can be expressed as:

(3) $$\begin{array}{c} p_{e}\left(\lambda |H_{0}\right)=\frac{\lambda^{\left(M-1\right)}e^{-\frac{\lambda }{\sigma_{n}^{2}}}}{\sigma_{n}^{2M}\Gamma \left(M\right)}, \end{array}$$

where $\lambda ={\left||y|\right|}_{l_{2}}^{2}$ is the energy of y.

If the coefficients in each single block of the measurement matrix are randomly selected from the identically independent complex Gaussian distributions and normalized to unit energy, the DSSS signal can also be modeled as AWGN over the DSSS spectrum in the signal detection scenario. Then in the signal present case, the theoretical PDF of the energy in an M-length measurement vector can be expressed as:

(4) $$\begin{array}{c} p_{e}\left(\lambda |H_{1}\right)=\frac{\lambda^{\left(M-1\right)}e^{-\frac{\lambda }{\left(\sigma_{n}^{2}+\sigma_{s}^{2}\right)}}}{{\left(\sigma_{n}^{2}+\sigma_{s}^{2}\right)}^{M}\Gamma \left(M\right)}\;, \end{array}$$

where $\sigma_{s}^{2}$ is the signal power.

The signal detection is done by energy thresholding. More specifically, given a threshold *T*, the theoretical false positive rate (FPR) and false negative rate (FNR) follow:

(5) $$\begin{array}{c} FPR=\int_{T}^{+\infty }p_{e}\left(\lambda |H_{0}\right)d\lambda \end{array}$$

and

(6) $$\begin{array}{c} FNR=\int_{-\infty }^{T}p_{e}\left(\lambda |H_{1}\right)d\lambda \;, \end{array}$$

respectively.

In this paper, we focus on adaptive knowledge-enhanced compressive measurements based on the TSI optimization. If we conduct the adaptations within symbol periods and design the measurement kernels for the measurements (i.e., the row blocks of the measurement matrix) sequentially, the measurement kernel for the *m*th measurement is designed by solving the following optimization problem:

(7) $$\begin{array}{c} \begin{array}{c} \begin{array}{cc} A_{m} & =arg\underset{A_{m}}{max}I\left(x_{m};y_{m}|{ℵ}_{m-1},A_{m}\right)\\ s.t.\;y_{m} & =A_{m}x_{m}\;\mathrm{and}\;||A_{m}{\left|\right|}_{l_{2}}=1\;, \end{array} \end{array} \end{array}$$

where ${ℵ}_{m-1}=\left\{A_{1},A_{2},\cdots ,A_{m-1},y_{1},y_{2},\cdots ,y_{m-1}\right\}$ is the collection of the measurement kernels and the measurement data in the 1st through the (m−1)th measurements. $A_{m}$, $x_{m}$ and $y_{m}$ represent the measurement kernel, preprocessed signal from the input filter and the measurement data at the *m*th measurement, respectively. ${∥\cdot ∥}_{l_{2}}$ represents the l–2 norm operation. The mutual information between $x_{m}$ and $y_{m}$, i.e., $I(x_{m};y_{m}|{ℵ}_{m-1},A_{m})$, is defined as the TSI in the signal detection.

During the operation period of $A_{m}$, if the channel noise and the DSSS signal in the signal present case are denoted as $n_{m}$ and $s_{m}$, then:

(8) $$\begin{array}{c} x_{m}=\left\{\begin{array}{ccc} \; & n_{m} & \;\mathrm{if}\;\mathrm{the}\;\mathrm{DSSS}\;\mathrm{signal}\;\mathrm{is}\;\mathrm{absent},\\ \; & s_{m}+n_{m} & \;\mathrm{if}\;\mathrm{the}\;\mathrm{DSSS}\;\mathrm{signal}\;\mathrm{is}\;\mathrm{present}. \end{array}\right. \end{array}$$

According to the information theory,

(9) $$\begin{array}{cc} I & \left(x_{m};y_{m}|{ℵ}_{m-1},A_{m}\right)\\ \; & =h\left(y_{m}|{ℵ}_{m-1},A_{m}\right)-h\left(y_{m}|{ℵ}_{m-1},A_{m},x_{m}\right)\;, \end{array}$$

where h(·|·) denotes the conditional differential entropy. If $s_{m}$ is known in the signal present case or in the signal absent case, the measurement data $y_{m}$ only depends on $A_{m}$ and the channel noise. Therefore, $h\left(y_{m}|{ℵ}_{m-1},A_{m},x_{m}\right)=h\left(y_{m}|A_{m},x_{m}\right)=h\left(A_{m}n_{m}\right)$. As the measurement noise $A_{m}n_{m}$ is a zero-mean circularly symmetric complex random variable with the variance of $\sigma_{n}^{2}$ according to the noise folding theory, $h(y_{m}|{ℵ}_{m-1},A_{m},x_{m})$ is a constant given the noise power. Thus, the optimization problem in Equation (7) is equivalent to:

(10) $$\begin{array}{c} \begin{array}{c} \begin{array}{cc} A_{m} & =arg\underset{A_{m}}{max}h\left(y_{m}|{ℵ}_{m-1},A_{m}\right)\\ s.t.\;y_{m} & =A_{m}x_{m}\;\mathrm{and}\;||A_{m}{\left|\right|}_{l_{2}}=1. \end{array} \end{array} \end{array}$$

In this paper, we focus on short-code DSSS (SC-DSSS) signals, where the period of the PN sequence is equal to the symbol period. In the case of measurements within symbol period, the measurement kernel $A_{m}$ is designed to cover at most the period of the PN sequence. To solve the statistical signal processing problems, the mixture of Gaussian (MoG) models has usually been used [35,36]. In measurement design stage of this paper, we establish a dictionary B of the DSSS signals. The atoms of the dictionary, denoted by $b_{l}$ (l=1,2,…,L), are taken to be the Nyquist rate sampled DSSS signals in a symbol period, which carry a fixed symbol content and are modulated by the possible PN sequences. Based on the dictionary, we establish a MoG model of the posterior distribution for the signal $s_{m}$ in the DSSS signal present case:

(11) $$\begin{array}{c} g\left(s_{m}|H_{1},{ℵ}_{m-1}\right)=\sum_{l=1}^{L}P_{b}\left(l|H_{1},{ℵ}_{m-1}\right)g_{l}\left(s_{m}\right), \end{array}$$

where *L* is the number of possible PN sequences, and $P_{b}\left(l|H_{1},{ℵ}_{m-1}\right)$ (1,2,…,L) denotes the posterior probability that the *l*th PN sequence is used in the DSSS signal present case, given the measurement kernels and data in the 1st through the (m−1)th measurements. The component $g_{l}\left(s_{m}\right)$ (1,2,…,L) is modeled with a complex zero-mean Gaussian distribution with the covariance matrix:

(12) $$\begin{array}{c} C_{\mathrm{ss}}^{\left(m,l\right)}=b_{l}^{\left(m\right)}{\left(b_{l}^{\left(m\right)}\right)}^{H}\;, \end{array}$$

where $b_{l}^{\left(m\right)}$ is a vector taken from the dictionary atom $b_{l}$, according to the locations of the coefficient block in the *m*th row of the measurement matrix. ${\left(\cdot \right)}^{H}$ represents the Hermitian operation.

With a simplified assumption that the single measurements are independent to each other, $P_{b}\left(l|H_{1},{ℵ}_{m-1}\right)$ (m>1) in Equation (11) can be obtained by:

(13) $$\begin{array}{c} P_{b}\left(l|H_{1},{ℵ}_{m-1}\right)=\frac{P_{b}\left(l|H_{1},{ℵ}_{m-2}\right)\frac{e^{-\frac{|y_{m-1}{|}^{2}}{\sigma_{l,m-1}^{2}}}}{\sigma_{l,m-1}^{2}}}{\sum_{l=1}^{L}P_{b}\left(l|H_{1},{ℵ}_{m-2}\right)\frac{e^{-\frac{|y_{m-1}{|}^{2}}{\sigma_{l,m-1}^{2}}}}{\sigma_{l,m-1}^{2}}}, \end{array}$$

where $\sigma_{l,m-1}^{2}$=$A_{m-1}C_{\mathrm{xx}}^{\left(m-1,l\right)}A_{m-1}^{H}$, with $C_{\mathrm{xx}}^{\left(m-1,l\right)}=C_{\mathrm{ss}}^{\left(m-1,l\right)}+\sigma_{n}^{2}E_{m-1}$. $E_{m-1}$ denotes the identity matrix in the same size of $C_{\mathrm{ss}}^{\left(m-1,l\right)}$.

With the MoG signal and AWGN channel models, if the rows of the compressive measurement matrix are normalized, it can be further proved that the signal absent case can be ignored in the optimization problem. Therefore, Equation (10) can be derived into the following form:

(14) $$\begin{array}{c} \begin{array}{c} \begin{array}{cc} A_{m} & =arg\underset{A_{m}}{max}h\left(y_{m}|H_{1},{ℵ}_{m-1},A_{m}\right)\\ s.t.\;y_{m} & =A_{m}\left(s_{m}+n_{m}\right)\;\mathrm{and}\;||A_{m}{\left|\right|}_{l_{2}}=1\;, \end{array} \end{array} \end{array}$$

where $h(y_{m}|H_{1},{ℵ}_{m-1},A_{m})$ is the conditional differential entropy of $y_{m}$ on $A_{m}$ in the signal present case, with the known measurement kernels and data in the 1st through the (m−1)th measurements. $h(y_{m}|H_{1},{ℵ}_{m-1},A_{m})$ can be approximated as:

(15) $$\begin{array}{c} h\left(y_{m}|H_{1},{ℵ}_{m-1},A_{m}\right)\approx -log\left[\sum_{l=1}^{L}\frac{P_{b}\left(l|H_{1},{ℵ}_{m-1}\right)}{\pi [A_{m}C_{\mathrm{xx}}^{\left(m,l\right)}A_{m}^{H}]}\right]\;, \end{array}$$

where $C_{\mathrm{xx}}^{\left(m,l\right)}=C_{\mathrm{ss}}^{\left(m,l\right)}+\sigma_{n}^{2}E_{m}$, with $E_{m}$ representing the identity matrix in the same size of $C_{\mathrm{ss}}^{\left(m,l\right)}$.

In the literature, to solve an optimization problem such as Equation (14), a recursive gradient method has usually been used [13,37]. In this method, the refinement of the measurement kernel $A_{m}$ at the *k*th iteration is performed using:

(16) $$\begin{array}{c} \begin{array}{c} \begin{array}{cc} {\bar{A}}_{m}^{\left(k\right)} & =A_{m}^{\left(k-1\right)}+\mu \nabla_{A_{m}}h\left(y_{m}|H_{1},{ℵ}_{m-1},A_{m}\right)\\ A_{m}^{\left(k\right)} & =\frac{{\bar{A}}_{m}^{\left(k\right)}}{\left|\right|{\bar{A}}_{m}^{\left(k\right)}{\left|\right|}_{l_{2}}}\;, \end{array} \end{array} \end{array}$$

where μ is the optimization step size, and the gradient item can be approximated as:

(17) $$\begin{array}{c} \begin{array}{c} \begin{array}{cc} \; & \nabla_{A_{m}}h\left(y_{m}|H_{1},{ℵ}_{m-1},A_{m}\right)\approx \\ \; & -\frac{\sum_{l=1}^{L}P_{b}\left(l|H_{1},{ℵ}_{m-1}\right){\left(A_{m}C_{\mathrm{xx}}^{\left(m\right)}A_{m}^{H}\right)}^{-2}A_{m}{C_{\mathrm{xx}}^{\left(m\right)}}^{H}}{\sum_{l=1}^{L}P_{b}\left(l|H_{1},{ℵ}_{m-1}\right){\left(A_{m}C_{\mathrm{xx}}^{\left(m\right)}A_{m}^{H}\right)}^{-1}}\;. \end{array} \end{array} \end{array}$$

The derivations to Equations (14), (15) and (17) are provided in the Appendix A.

## 4. Adaptive Compressive Detection of Direct Spread-Spectrum Signals with the Artificial Neural Network

### 4.1. Design of the Artificial Neural Networks

To get the convergence of the recursive method described in Equation (16) and achieve improved detection performance over the conventional compressive measurement method with random kernels, usually thousands of recursive steps are needed for one single measurement design. This can be extremely time-consuming. In the online adaptive measurement scenario, the low efficiency of the recursive optimization method makes its implementations infeasible.

**Figure 2:**
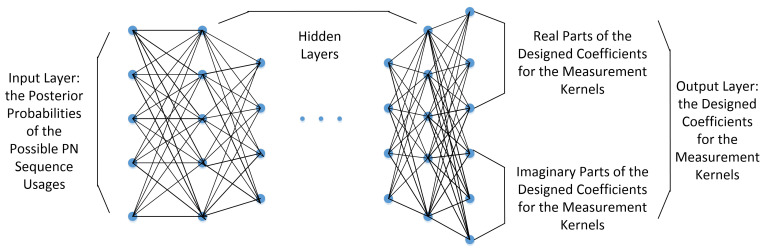
The Architecture of the Artificial Neural Networks in the Adaptive Measurement Kernel Design.

To improve the algorithm efficiency, we propose to implement the artificial neural networks to conduct the adaptive measurement kernel design. The architecture of the neural networks is described in Figure 2.

In Figure 2, the nodes of the input layer represent the posterior probabilities of the possible PN sequence usages in the signal present case, given the 1st through the (m−1)th measurements, i.e., $P_{b}\left(l|H_{1},{ℵ}_{m}\right)$ (1,2,…,L) in Equation (11). The neural network is fully connected and real valued. The nodes of the output layer hold the designed coefficients for the measurement kernels. Considering that the coefficients in the measurement kernels are complex valued, half of the nodes in the output layer represent their real parts, while the other nodes represent their imaginary parts. The resulting coefficients from the output layer are used in the measurement matrix, where the rows are further normalized to unit energy.

In this paper, two neural network strategies are proposed for the adaptive measurement kernel design. In the first strategy, independent neural networks are trained to design the measurement kernels for different measurements in a single symbol period, respectively. Thus, the number of neural networks to be trained is equal to the number of measurements for a symbol period. In the second strategy, a single neural network that can be used to get the measurement kernels for all the measurements in a symbol period is designed. In this case, only a part of the designed coefficients are used for each measurement. To be concise, the two neural network strategies above are referred to as “multiple neural network strategy” and “single neural network strategy” in the remainder of this paper.

The training of the neural networks follows the gradient back-propagation algorithm. The training data are the randomly generated usage probability vectors of the possible PN sequences. To train the neural network to get the measurement kernel of the *m*th measurement in the multiple neural network strategy, the training penalty function is taken to be the negativity of the conditional differential entropy in Equation (15), i.e., $-h(y_{m}|H_{1},{ℵ}_{m-1},A_{m})$. Such a penalty function depends on the posterior probabilities of the PN sequence usage and the designed measurement kernel, i.e., $P_{b}\left(l|H_{1},{ℵ}_{m-1}\right)$ and $A_{m}$, according to Equation (15). In the single neural network strategy, the training penalty function is then taken to be $-\sum_{m=1}^{M}h\left(y_{m}|H_{1},{ℵ}_{m-1},A_{m}\right)$.

In contrast to the recursive measurement kernel optimization method discussed in Section 2, the artificial neural networks proposed in this paper is trained once off-line and implemented in the online adaptive measurements efficiently.

### 4.2. The Procedure of the Adaptive Measurement and Detection of Direct-Sequence Spread-Spectrum Signals Using the Artificial Neural Network

**Figure 3:**
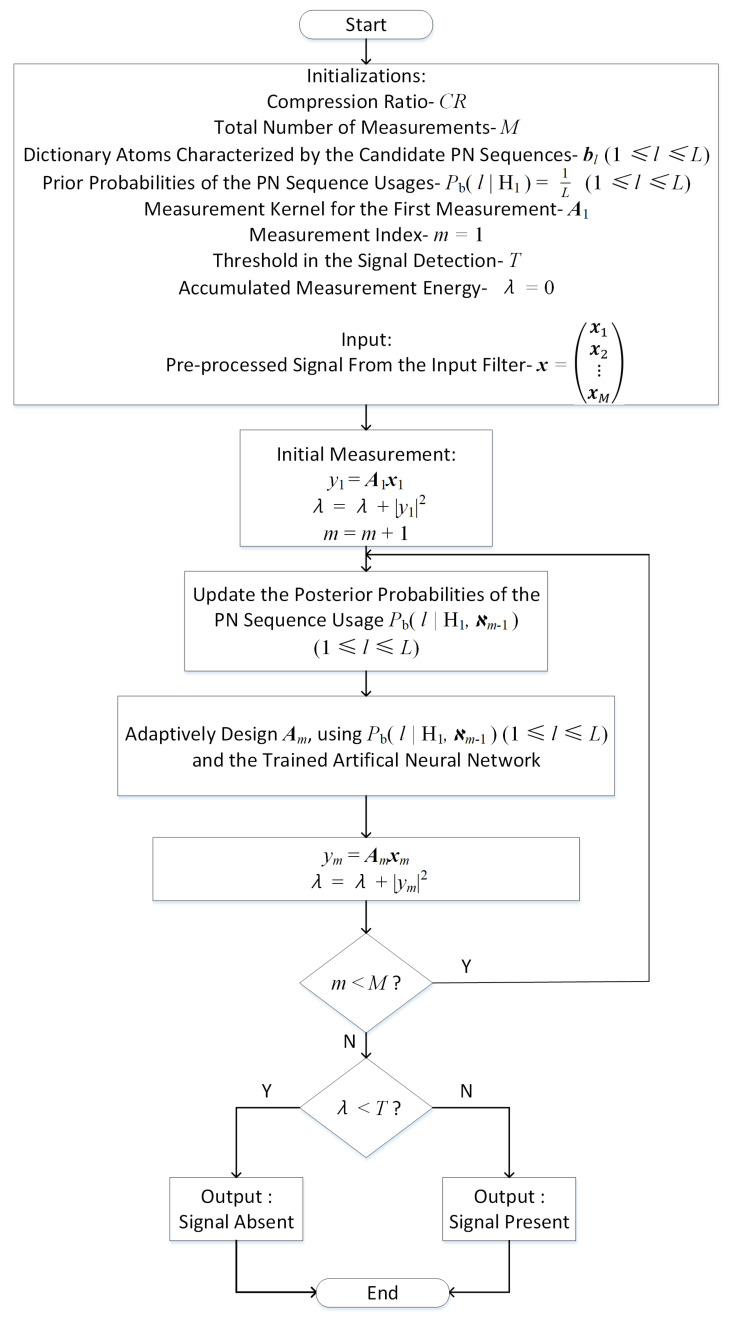
The Proposed Adaptive Measurement and Detection Procedure of the DSSS Signals.

With the combined operations of the adaptive compressive framework shown in Figure 1 and the artificial neural networks described in Figure 2, the adaptive measurement and detection procedure of the DSSS signals can be described in Figure 3.

In Figure 3, the non-zero coefficients of the measurement matrix for initial measurement are generated according to the identically independent complex Gaussian distributions and then normalized to unit energy. With equal prior probabilities of usages for the possible PN sequences, the posterior probabilities are then recursively updated with the measurement kernels and data, according to Equation (13). Meanwhile, the measurement kernels are adaptively designed using the neural networks.

The energy of the resulting measurement data from the entire procedure, i.e., λ, is finally collected for signal detection. The detection of the DSSS signals is done based on the measurement energy thresholding, as described in Section 3.

It is worth mentioning that although the adaptive measurements in the above discussions are proposed within single symbol-period scale, the measurement procedure can be extended across multiple symbol periods, depending on the number of measurements needed to make the detection decision. The detection performance of this extension is also simulated and discussed in Section 5.

## 5. Evaluations and Discussions through Theoretical Analysis and Simulations

**Figure 4:**
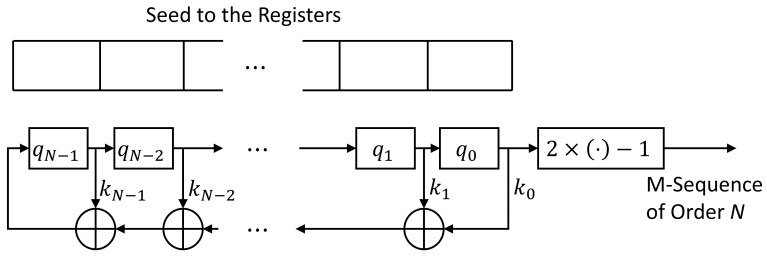
Structure of the Feedback Shift-Registers to Generate the M-Sequences.

In this paper, we used the binary PSK (BPSK) modulated SC-DSSS signals in the theoretical analysis and simulations. The possible candidate PN sequences were taken from the maximum-length sequences (m-sequences) [19,38] of the orders 1 through 5, which were commonly implemented in DSSS communications. The m-sequences at the order *N* were generated with the feedback shift-registers with the structure described in Figure 4.

In Figure 4, a seed to the shift-registers is a binary sequence at the length *N*, where not all the entries are zero-valued. $q_{0},q_{1},\ldots q_{N-1}\in \left\{0,1\right\}$ are the values stored in the registers. The binary multipliers $k_{0},k_{1},\ldots k_{N-1}\in \left\{0,1\right\}$ are generated from the primitive polynomials $k_{0}+k_{1}x+\ldots +k_{N-1}x^{N-1}$. The additions in Figure 4 are binary additions, and the module at the end of registers coverts the binary {0,1} to the values in {−1,1}. The primitive polynomials ordered from 1 to 5 and the number of the m-sequences are shown in Table 1.

The maximum length of the m-sequences specified in Table 1 is 31. Therefore, we took the number of Nyquist samples from each symbol period as 62 in the theoretical analysis and the simulations.

Both multiple neural network strategy and the single neural network strategy described in Section 4 were performed in this section. 3 hidden layers were included for each of the neural networks trained in this paper. For the single neural network strategy, the widths of the 3 hidden layers were 350, 128 and 64, respectively. For the multiple neural network strategy, the hidden layer widths were taken to be 512, 350, 256, respectively.

The neural networks were optimized using the TensorFlow 2.0 GPU version [39] based on Python 3.7. To train each of the neural networks, 20,000 random probability vectors (at the batch size of 100 and 10 epochs) were used as the training data. The resulting neural networks were used to evaluate the performance of the proposed adaptive methods.

In the simulations, we define the SNR as the ratio between the signal power and the noise variance, i.e.,

(18) $$SNR=\frac{\sigma_{s}^{2}}{\sigma_{n}^{2}}$$

As is discussed in Section 4, the measurements and detections can be done within single symbol period or across multiple symbol periods. In the remainder of this section, we first evaluate theoretical analysis and simulated performance of the proposed adaptive measurement and detection methods on single symbol-period basis. Then, the simulated results with the measurements and detections across multiple symbol periods are provided and discussed as an extension to the theory discussed in Section 3 and Section 4.

### 5.1. The Theoretical Analysis and Simulations of DSSS Detection through Single Symbol-Period Measurements

As is specified above, in the theoretical analysis and simulations of the measurements and detection within single symbol period, the number of Nyquist samples was taken to be 62. To conduct compressive measurements, the number of compression measurements for detection were chosen from 6, 9 and 12 in this part, resulting in the CR values of about 10, 7 and 5, respectively.

**Figure 5:**
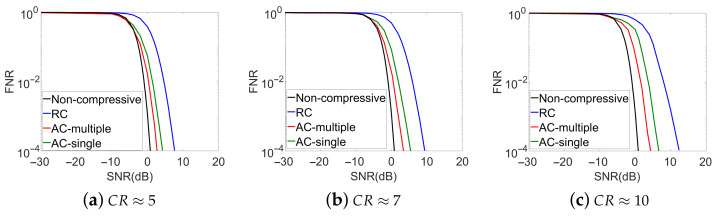
Theoretical Analysis of the Detection Accuracies from Single Symbol-Period Measurements (Non-compressive: Non-compressive detection method; RC: Conventional compressive detection with random measurement kernels; AC-multiple: Adaptive compressive detection with the multiple neural network strategy; AC-single: Adaptive compressive detection with the single neural network strategy).

We first analyzed the theoretical detection accuracies of the proposed methods through single symbol-period measurements. As the adaptive measurement processes are stochastic with feedbacks, it is difficult to analyze their theoretical detection performance with closed formulas. To surrogate, we took an approximation, where the PN sequence used in the DSSS signals was exactly known in each detection and the posterior probabilities of the PN sequence usage in each measurement kernel adaptation were given as a binary 1-sparse vector. In this case, we ran Monte-Carlo simulations with the SNR values ranging from −30 dB to 20 dB. The curves of the FNR versus the SNR are plotted in Figure 5 for the 3 CR cases, where each point in the curves was generated using 100,000 simulations. In each simulation, the PN sequence were selected randomly from the 234 possible candidates with equal probabilities. The detection thresholds were obtained with the theoretical FPRs to be 0.01, according to Equations (3) and (5). Consequentially, the curves of the proposed adaptive methods in Figure 5 represent their best possible results and are regarded as their theoretically analyzed detection accuracy results.

For comparison, the theoretical performance of the non-compressive energy detection method and the conventional compressive detection method with random measurement kernels at the 3 CR values were also analyzed according to Equations (3)–(6). The analyzed results are shown in Figure 5. In the non-compressive energy detection, the measurement matrix was an identity matrix, thus no compression was done during the measurements and the number of measurements was equal to the number of Nyquist samples. In the conventional compressive detection method, the coefficient blocks of each row in the measurement matrix were randomly selected from the identically independent complex zero-mean Gaussian distributions and then normalized to unit energy. The thresholds used in these two methods were also obtained by taking their theoretical FPRs as 0.01.

From Figure 5, we observe that the detection accuracies for all the methods generally improve with decreased CR values. This improvement is resulted from the more and more distinguished statistics of the measurement energies between the signal absent and present cases. The non-compressed method gets the best detection accuracy and can be treated as a benchmark. Comparing the detection accuracies of the compressive methods, we observe that the theoretical optimal performance of the proposed methods are significantly improved over the conventional compressive detection method with random measurement kernels. For example, to achieve a given FNR value at CR≈ 5, the proposed methods can save up to about 5 dB in SNR at their theoretical best performance, compared to the conventional compressed detection system with the random measurement kernel.

For the proposed adaptive methods, if we compare the multiple neural network and single neural network strategies, we observe that the adaptive method with the multiple neural network strategy shows slightly better performance in the detection accuracy than the adaptive method with single neural network strategy. As a trade-off, a higher cost in the hardware and network training time is introduced by the multiple neural network strategy.

**Figure 6:**
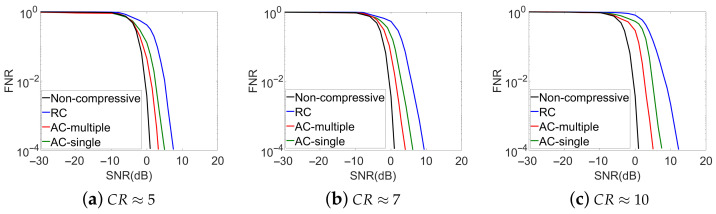
Simulated Detection Accuracies from Single Symbol-Period Measurements. (Non-compressive: Non-compressive detection method; RC: Conventional compressive detection with random measurement kernels; AC-multiple: Adaptive compressive detection with the multiple neural network strategy; AC-single: Adaptive compressive detection with the single neural network strategy.)

Besides the theoretical analysis, the Monte-Carlo simulations of the DSSS signals detection using the proposed adaptive methods were also performed for the 3 CR cases. The system setups were similar to the theoretical analysis. The simulated FNR results versus SNR for the proposed methods are shown in Figure 6. To generate each point in these curves, 5,000,000 Monte-Carlo simulations were done. The simulation results of non-compressive detection method and conventional compressive detection method with random measurement kernels are also shown in Figure 6 for comparisons. Similar to the theoretical analysis, the thresholds used in the detection step were generated according to Equations (3) and (5), with the theoretical FPRs to be 0.01.

Comparing Figure 5 and Figure 6, we observe that the simulated performance of the non-compressive detection method and the conventional compressive detection method with random measurement kernels match well with their theoretically analyzed results. The simulated detection accuracies of the proposed methods, although are slightly lower than their theoretical optimum cases at given SNR and CR values sometimes, are still significantly improved compared to the conventional compressive method with random measurement kernels. In addition, we can see that the signal can also be detected even when the SNR is lower than 0 dB. This is because that the designed measurement kernels concentrated more and more on the signal as the adaptive measurements proceeded, which leads to the increased SNRs in the measurement data.

To validate the discussions above and have a deeper insight into the proposed adaptive methods, we conducted a further study on the correlations between the rows of the designed measurement matrix and the PN sequence that was factually used in the DSSS signal generation. In this paper, the correlation between the *m*th row of the measurement matrix and the used PN sequence (assuming that the *v*th PN sequence was factually used) is defined by:

(19) $$\xi_{m}=\frac{|<A_{m},b_{v}>|}{∥A_{m}{∥}_{l_{2}}\cdot {∥b_{v}∥}_{l_{2}}}\;$$

where <·,·> and |·| denote the inner product operations and the absolute value, respectively. $A_{m}$ is the *m*th row of the measurement matrix A, and $b_{v}$ represents the *v*th dictionary atom discussed in the MoG model in Section 3. A larger correlation value from Equation (19) indicates a higher SNR in the measurement result, which in turn leads to a higher detection accuracy.

**Figure 7:**
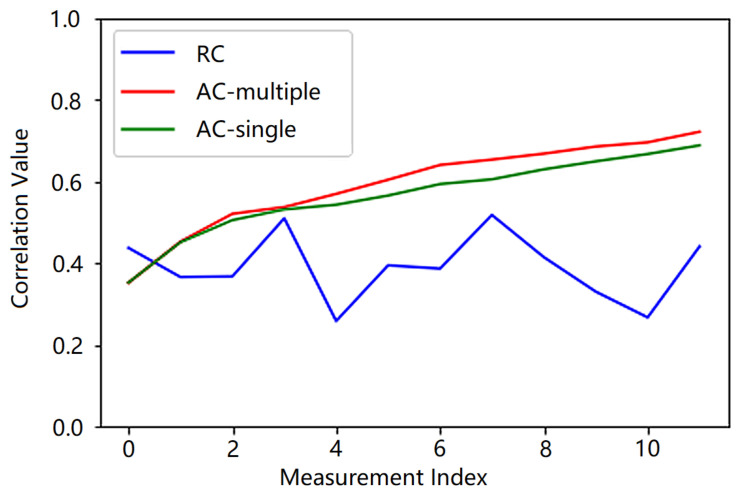
Correlations between the Compressive Measurement Kernels and the Used PN Sequence (RC: Conventional compressive detection with random measurement kernels; AC-multiple: Adaptive compressive detection with the multiple neural network strategy; AC-single: Adaptive compressive detection with the single neural network strategy).

As a representative, Figure 7 depicts the correlation values versus the measurement indices in a symbol-period adaptive procedure for the proposed adaptive methods at CR≈ 5 and SNR=−10 dB. To compare, the curve for the conventional compressive detection method with random measurement kernels is also shown in Figure 7. To generate each point in the curves, 100,000 Monte-Carlo simulations were done, where the PN sequence used in each simulation were randomly selected from the 234 possible candidates with equal probabilities, and the resulting correlation values at each SNR were averaged.

In Figure 7, it can be observed that the correlation values gradually increase for the proposed adaptive methods, as the measurements proceed. This indicates that the designed measurement kernels concentrate more and more on the signal as the adaptive measurements proceed, leading to gradually increased SNRs in the measurement data and the improved detection accuracies. In contrast, for the random measurement kernels, the correlation values randomly fluctuate around the value of 0.4 and are lower than those of the proposed adaptive case over almost the entire measurement procedure. Thus, the SNR of the measurement data is relatively lower for conventional compressive detection method, which in turn results in a lower detection accuracy. Comparing the curves of the two proposed neural network strategies, we find that the correlation value from the multiple neural network strategy increases slightly faster than that from the single neural network strategy. This in turn results in slightly improved detection accuracy than the single neural network strategy.

Besides the studies on the detection accuracies and the measurement procedures of the proposed adaptive methods, we also conducted a study on the time costs of the proposed adaptive measurement kernel design methods based on artificial neural networks to observe their efficiencies. To validate, the time cost of the recursive optimization method described in Section 3 was also observed for comparison. For quantitative evaluations, the time costs of the measurement kernel design for 500 measurements (i.e., the time costs for the adaptive measurements over 100 single symbol-period detections) were measured with the proposed methods and the recursive optimization method at CR ≈ 10 and SNR=4 dB. For the recursive optimization method, to reach the detection accuracies using the artificial neural networks, 2000 iterations are usually needed to design the measurement kernel for a single method, which was implemented in this study. The simulations were done on a computer with the CPU of Intel Core i5-9400 @ 2.90 GHz and the RAM size of 32.00 GB. The timing information of the measurement kernel design for a single measurement are shown in Table 2.

From Table 2, it can be seen that the efficiencies of the proposed methods are significantly improved over the recursive optimization method in the measurement kernel design. The improvement can be as high as around 10,000 times. Comparing the two strategies in the proposed methods, the multiple neural network strategy results in slightly lower time cost, as the structure of each neural network in this strategy is relatively simpler. Although the time costs of the proposed methods shown in Table 2 are still relatively high for the practical DSSS signals detection, the efficiency can be expected to be significantly improved with the specially designed hardware and software. This improvement will be studied in our future work.

### 5.2. The Simulations of DSSS Detection through Multi-Symbol-Period Measurements

**Figure 8:**
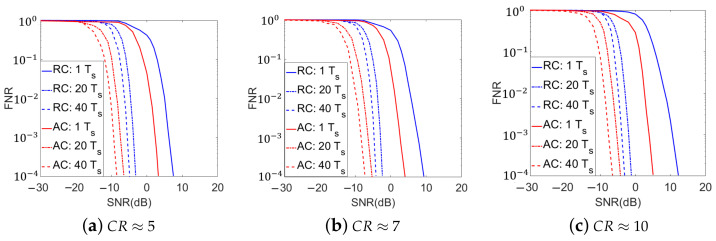
Simulated Detection Accuracies over Multiple Symbol Periods with the Multiple Neural Network Strategy for the Proposed Adaptive Method (RC: Conventional compressive detection with random measurement kernels; AC: Adaptive compressive detection with the multiple neural network strategy. $T_{s}$ represents the time of one symbol period).

**Figure 9:**
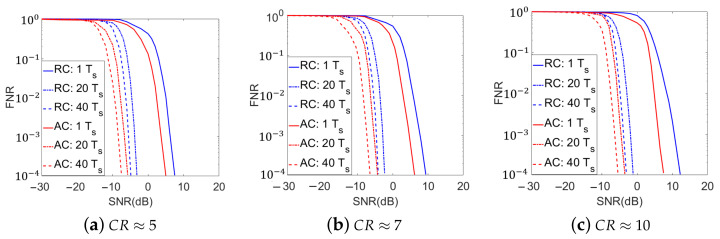
Simulated Detection Accuracies over Multiple Symbol Periods with the Single Neural Network Strategy for the Proposed Adaptive Method (RC: Conventional compressive detection with random measurement kernels; AC: Adaptive compressive detection with the single neural network strategy. $T_{s}$ represents the time of one symbol period).

It has been discussed in Section 4 that the proposed adaptive methods can be extended for DSSS signals detection with the measurements over multiple symbol periods. In this paper, with similar setups to those in the single symbol-period detection simulations, we also conducted Monte-Carlo simulations for the DSSS signals detection over on multi-symbol-period measurements. In these simulations, the coefficients in the measurement kernel for the first measurement of the first symbol period were generated according to identically independent complex zero-mean Gaussian distributions and then normalized. Then, the measurement kernels for the other measurements are sequentially designed. The adaptations within each symbol period were done similarly to single symbol period simulations. For inter-symbol adaptations, the measurement kernels corresponding to the first measurements in the 2nd though the last symbol periods were adaptively designed based on the posterior information from measurements in the previous symbol periods. The simulated curves of the FNR versus the SNR for the 3 CR values are shown in Figure 8 and Figure 9, which corresponds to the multiple neural network and single neural network strategies, respectively. The numbers of symbol periods included in the entire measurement procedure were selected as 20 and 40. To compare, the simulated results of the conventional compressive method with random kernels over single and multiple symbol periods, as well as the proposed adaptive methods with single symbol-period measurements, are also plotted in Figure 8 and Figure 9.

In Figure 8 and Figure 9, we find that at any given CR value, the multi-symbol-period implementations of the proposed adaptive methods get better detection accuracies than the conventional compressive method with random measurement kernels, which is similar to the single symbol-period detection. For example, at CR ≈ 5 and SNR=−6 dB, the conventional compressive detection method with 20 symbol periods yields an FNR of 0.1244 and the adaptive method with the single neural network strategy gets the FNR lower than 0.001, which is about 100 times’ improvement. We also observe that the multi-symbol-period signal detection performance of all systems, especially for the proposed adaptive methods, gets improved over those of their single symbol-period implementations. In particular, the more symbol periods that are included in the measurements, the better signal detection accuracies the systems can achieve. For example, at CR≈5 and SNR=−6 dB, the adaptive method using the multiple neural network strategy in single symbol period yields an FNR of 0.7432, while the resulting FNRs of the adaptive methods over 20 and 40 symbol periods are lower than 0.001 and 0.0001, which are about 740 times’ and 7400 times’ improvement, respectively. Similar to the single symbol-period detection simulations, with higher hardware and training time costs, the adaptive method with the multiple neural network strategy also shows slightly better performance than the single neural network strategy for multi-symbol-period implementations.

For the simulation results in Figure 8 and Figure 9, besides the increased number of measurements that makes the energy statistics in the signal absent and present cases get more and more distinguished, the gradually increased correlation between the designed measurement kernels and the signal (as more posterior information updates and measurement kernel adaptations are done in this scenario) in the proposed adaptive methods also plays an important role in the detection accuracy improvement. On the other hand, the time cost in the detection task is increased in this scenario. Therefore, in practical implementations, the trade-off between the time cost and the detection accuracy needs to be comprehensively considered, according to the detailed detection tasks.

## 6. Conclusions

In this paper, we proposed adaptive methods to measure and detect the DSSS signals using knowledge-enhanced compressive measurements. The detection was done based on energy detection and the measurement matrix was designed adaptively based on TSI optimization. To improve the measurement design efficiency and make the system feasible, the artificial neural networks were trained and implemented in the adaptive measurement kernel design, as a surrogate of the recursive optimization method in the literature. Theoretical analysis and simulations were performed to compare the proposed methods, the non-compressive detection method and the conventional compressive detection method using random measurement kernels. The theoretical and simulation results demonstrated that the proposed methods provided significantly enhanced detection accuracies with high efficiency, compared to the conventional compressive detection method with random measurement kernels.

## Figures and Tables

**Table 1:** Primitive Polynomials and the Numbers of M-Sequences Ordered from 1 to 5.

Order	Primitive Polynomials	Number of M-Sequences at This Order	Total Number of M-Sequences Ordered 1–5
1	1 + *x*	1 × 1 = 1	
2	1 + *x* + $x^{2}$	3 × 1 = 3	
3	1 + *x* + $x^{3}$ 1 + $x^{2}$ + $x^{3}$	7 × 2 = 14	
4	1 + *x* + $x^{4}$ 1 + $x^{3}$ + $x^{4}$	15 × 2 = 30	234
5	1 + $x^{2}$ + $x^{5}$ 1 + *x* + $x^{2}$ + $x^{3}$ + $x^{5}$ 1 + $x^{3}$ + $x^{5}$ 1 + *x* + $x^{3}$ + $x^{4}$ + $x^{5}$ 1 + $x^{2}$ + $x^{3}$ + $x^{4}$ + $x^{5}$ 1 + *x* + $x^{2}$ + $x^{4}$ + $x^{5}$	31 × 6 = 186	

**Table 2:** Time Cost Information of the Measurement Kernel Design for a Single Measurement.

Method	Minimum Time Consumed	Average Time Consumed	Maximum Time Consumed
Recursive Optimization	4610.2430 s	4724.1410 s	4902.9858 s
Multiple Neural Network Strategy	0.4119 s	0.4324 s	0.4428 s
Single Neural Network Strategy	0.4328 s	0.4487 s	0.4543 s

## Abbreviations

The following abbreviations are used in this manuscript:

DSSS	Direct-sequence spread-spectrum
SS	Spread-spectrum
PN	Pseudo-noise
CS	Compressive sensing
TSI	Task-specific information
CR	Compression ratio
PSK	Phase-shift-keying
AWGN	Additive white Gaussian noise
PDF	Probability density function
FPR	False positive rate
FNR	False negative rate
SC-DSSS	Short-code DSSS
MoG	Mixture of Gaussian
BPSK	binary PSK
M-sequence	maximum-length sequence
SNR	signal-to-noise ratio

## Appendix A

In the Appendix A, we provide the details of the derivations used to obtain Equations (14), (15) and (17). Recall that we established a MoG model for the signal $s_{m}$ (*m* ≤ 1), which was defined as the noise-free DSSS signal measured by the measurement kernel in the *m*th row of the measurement matrix in a symbol period in the signal present case. In this scenario, as the channel noise is also modeled with Gaussian distributions, then given the measurement kernel $A_{m}$, the distribution of the *m*th measurement in that symbol period, i.e., $y_{m}$, is also an MoG distribution.

(A1) $$\begin{array}{cc} \; & f(y_{m}|{ℵ}_{m-1},A_{m})\\ = & P_{H}\left(H_{1}|{ℵ}_{m-1}\right)\sum_{l=1}^{L}P_{b}\left(l|H_{1},{ℵ}_{m-1}\right)f_{l}\left(y_{m}|A_{m}\right)+P_{H}\left(H_{0}|{ℵ}_{m-1}\right)f_{0}\left(y_{m}|A_{m}\right)\;, \end{array}$$

where $P_{H}\left(H_{0}|{ℵ}_{m-1}\right)$ and $P_{H}\left(H_{1}|{ℵ}_{m-1}\right)$ denotes the probability of the signal absent and signal present cases, respectively, given the measurement kernels and data in the 1st through the (m−1)th measurements (i.e., ${ℵ}_{m-1}$). $P_{b}\left(l|H_{1},{ℵ}_{m-1}\right)$ denotes the probability that the *l*th PN sequence is used in the signal present case, given the measurement kernels and data in the 1st through the (m−1)th measurements. The Gaussian components of $y_{m}$ in the signal absent case and present case with the *l*th PN sequence used are represented by:

(A2) $$f_{0}\left(y_{m}|A_{m}\right)=\mathrm{CN}\left(0,\sigma_{n}^{2}A_{m}E_{m}A_{m}^{H}\right)$$

and

(A3) $$f_{l}\left(y_{m}|A_{m}\right)=\mathrm{CN}\left(0,A_{m}C_{xx}^{\left(m,l\right)}A_{m}^{H}\right)\;,$$

where CN(·,·) denotes the complex Gaussian PDF, with the first and second parameters representing the mean and the variance of the Gaussian component, respectively. $\sigma_{n}^{2}$ is the noise variance. $C_{xx}^{\left(m,l\right)}=C_{ss}^{\left(m,l\right)}+\sigma_{n}^{2}E_{m}$, where $C_{ss}^{\left(m,l\right)}$ and $E_{m}$ are the covariance matrix of $s_{m}$ with the *l*th PN sequence used in the signal present case and the identity matrix with the size of $C_{ss}^{\left(m,l\right)}$, respectively.

According to the information theory and the MoG distribution above, the conditional differential entropy item $h(y_{m}|{ℵ}_{m-1},A_{m})$ on $A_{m}$ with certain ${ℵ}_{m-1}$ can be derived as follows:

(A4) $$\begin{array}{cc} \; & h\left(y_{m}|{ℵ}_{m-1},A_{m}\right)=-\int f\left(y_{m}|{ℵ}_{m-1},A_{m}\right)log\left[f(y_{m}|{ℵ}_{m-1},A_{m})\right]dy_{m}\\ = & -\int f(y_{m}|{ℵ}_{m-1},A_{m})\\ \; & \cdot log\left[P_{H}\left(H_{1}|{ℵ}_{m-1}\right)\sum_{l=1}^{L}P_{b}\left(l|H_{1},{ℵ}_{m-1}\right)f_{l}\left(y_{m}|A_{m}\right)+P_{H}\left(H_{0}\right|{ℵ}_{m-1}\right)f_{0}\left(y_{m}|A_{m}\right)]dy_{m}\;. \end{array}$$

If we approximate the logarithm item in Equation (A4) by the first two terms of its Taylor expansion at $y_{m}=0$, we get:

(A5) $$\begin{array}{cc} \; & log\left[P_{H}\left(H_{1}|{ℵ}_{m-1}\right)\sum_{l=1}^{L}P_{b}\left(l|H_{1},{ℵ}_{m-1}\right)f_{l}\left(y_{m}|A_{m}\right)+P_{H}\left(H_{0}|{ℵ}_{m-1}\right)f_{0}\left(y_{m}|A_{m}\right)\right]\\ = & log\left[P_{H}\left(H_{1}|{ℵ}_{m-1}\right)\sum_{l=1}^{L}P_{b}\left(l|H_{1},{ℵ}_{m-1}\right)f_{l}\left(0|A_{m}\right)+P_{H}\left(H_{0}|{ℵ}_{m-1}\right)f_{0}\left(0|A_{m}\right)\right]+\Xi \left(0\right)y_{m}\\ \; & +H.O.T\\ \approx & log\left[P_{H}\left(H_{1}|{ℵ}_{m-1}\right)\sum_{l=1}^{L}P_{b}\left(l|H_{1},{ℵ}_{m-1}\right)f_{l}\left(0|A_{m}\right)+P_{H}\left(H_{0}|{ℵ}_{m-1}\right)f_{0}\left(0|A_{m}\right)\right]+\Xi \left(0\right)y_{m}\;, \end{array}$$

where

(A6) $$\begin{array}{cc} \Xi (0)= & \nabla_{y_{m}}\{log[P_{H}\left(H_{1}|{ℵ}_{m-1}\right)\sum_{l=1}^{L}P_{b}\left(l|H_{1},{ℵ}_{m-1}\right)f_{l}\left(y_{m}|A_{m}\right)\\ \; & +P_{H}\left(H_{0}|{ℵ}_{m-1}\right)f_{0}\left(y_{m}|A_{m}\right)]\}{|}_{y_{m}=0} \end{array}$$

and H. O. T denotes the higher order terms in the Taylor expansion.

Substituting Equations (A5) and (A6) into Equation (A4), we can get:

(A7) $$\begin{array}{cc} \; & h\left(y_{m}|{ℵ}_{m-1},A_{m}\right)\approx -\int f\left(y_{m}|{ℵ}_{m-1},A_{m}\right)\{log[P_{H}\left(H_{1}|{ℵ}_{m-1}\right)\sum_{l=1}^{L}P_{b}\left(l|H_{1},{ℵ}_{m-1}\right)f_{l}\left(0|A_{m}\right)\\ \; & +P_{H}\left(H_{0}|{ℵ}_{m-1}\right)f_{0}\left(0|A_{m}\right)]+\Xi \left(0\right)y_{m}\}dy_{m}\\ = & -log\left[P_{H}\left(H_{1}|{ℵ}_{m-1}\right)\sum_{l=1}^{L}P_{b}\left(l|H_{1},{ℵ}_{m-1}\right)f_{l}\left(0|A_{m}\right)+P_{H}\left(H_{1}|{ℵ}_{m-1}\right)f_{0}\left(0|A_{m}\right)\right]\\ = & -log\left[P_{H}\left(H_{1}|{ℵ}_{m-1}\right)\sum_{l=1}^{L}\frac{P_{b}\left(l|H_{1},{ℵ}_{m-1}\right)}{\pi (A_{m}C_{\mathrm{xx}}^{\left(m,l\right)}A_{m}^{H})}+\frac{P_{H}\left(H_{0}|{ℵ}_{m-1}\right)}{\pi (\sigma_{n}^{2}A_{m}E_{m}A_{m}^{H})}\right]\;. \end{array}$$

The posterior probabilities $P_{H}\left(H_{0}|{ℵ}_{m-1}\right)$ and $P_{H}\left(H_{1}|{ℵ}_{m-1}\right)$ are constants, when the measurement kernels and data from the 1st through the (m−1)th measurements are known. Meanwhile, the rows of the compressive measurement matrix are normalized, i.e., $\left|\right|A_{m}{\left|\right|}_{l_{2}}$ = 1. Thus, the second term in result of Equation (A7) is also a constant, and the optimization problem in Equation (10) is equivalent to the maximization of conditional differential entropy $h(y_{m}|H_{1},{ℵ}_{m-1},A_{m})$ as follows:

(A8) $$h\left(y_{m}|H_{1},{ℵ}_{m-1},A_{m}\right)\approx -log\left[\sum_{l=1}^{L}\frac{P_{b}\left(l|H_{1},{ℵ}_{m-1}\right)}{\pi (A_{m}C_{\mathrm{xx}}^{\left(m,l\right)}A_{m}^{H})}\right]\;.$$

Using the chain rule of the gradient operation, we can then obtain the gradient of $h(y_{m}|H_{1},{ℵ}_{m-1},A_{m})$ in Equation (A8):

(A9) $$\begin{array}{cc} \; & \nabla_{A_{m}}h\left(y_{m}|H_{1},{ℵ}_{m-1},A_{m}\right)\approx \nabla_{A_{m}}\left\{-log\left[\sum_{l=1}^{L}\frac{P_{b}\left(l|H_{1},{ℵ}_{m-1}\right)}{\pi (A_{m}C_{\mathrm{xx}}^{\left(m,l\right)}A_{m}^{H})}\right]\right\}\\ \; & =-\frac{\sum_{l=1}^{L}P_{b}\left(l|H_{1},{ℵ}_{m-1}\right)\pi^{-1}\nabla_{A_{m}}\left\{{\left(A_{m}C_{\mathrm{xx}}^{\left(m,l\right)}A_{m}^{H}\right)}^{-1}\right\}}{\sum_{l=1}^{L}P_{b}\left(l|H_{1},{ℵ}_{m-1}\right)\pi^{-1}{\left(A_{m}C_{\mathrm{xx}}^{\left(m,l\right)}A_{m}^{H}\right)}^{-1}}\\ \; & =-\frac{\sum_{l=1}^{L}P_{b}\left(l|H_{1},{ℵ}_{m-1}\right){\left(A_{m}C_{\mathrm{xx}}^{\left(m,l\right)}A_{m}^{H}\right)}^{-2}A_{m}{C_{\mathrm{xx}}^{\left(m,l\right)}}^{H}}{\sum_{l=1}^{L}P_{b}\left(l|H_{1},{ℵ}_{m-1}\right){\left(A_{m}C_{\mathrm{xx}}^{\left(m,l\right)}A_{m}^{H}\right)}^{-1}}\;. \end{array}$$
